# The effects of mindfulness-based cognitive therapy on risk and protective factors of depressive relapse – a randomized wait-list controlled trial

**DOI:** 10.1186/s40359-020-00417-1

**Published:** 2020-06-05

**Authors:** Elisabeth Schanche, Jon Vøllestad, Endre Visted, Julie Lillebostad Svendsen, Berge Osnes, Per Einar Binder, Petter Franer, Lin Sørensen

**Affiliations:** 1 Department of Clinical Psychology, University of Bergen, Bergen, Norway; 2 Solli District Psychiatric Centre (DPS), Nesttun, Norway; 3 Kronstad District Psychiatric Centre (DPS), Division of Psychiatry, Haukeland University Hospital, Bergen, Norway; 4 Department of Biological and Medical Psychology, University of Bergen, Bergen, Norway; 5 Bjørgvin District Psychiatric Centre (DPS), Division of Psychiatry, Haukeland University Hospital, Bergen, Norway

**Keywords:** MBCT, Recurrent depression, Emotion regulation, Risk factors of depressive relapse, Protective factors of depressive relapse

## Abstract

**Background:**

The aim of this randomized wait-list controlled trial was to explore the effects of Mindfulness–Based Cognitive Therapy (MBCT) on risk and protective factors for depressive relapse within the domains of cognition, emotion and self-relatedness.

**Methods:**

Sixty-eight individuals with recurrent depressive disorder were randomized to MBCT or a wait-list control condition (WLC).

**Results:**

Completers of MBCT (*N* = 26) improved significantly on measures assessing risk and protective factors of recurrent depression compared to WLC (*N* = 30) on measures of rumination (*d* = 0.59, *p* = .015), emotion regulation (*d* = 0.50, *p* = .028), emotional reactivity to stress (*d* = 0.32, *p* = .048), self-compassion (*d* = 1.02, *p* < .001), mindfulness (*d* = 0.59, *p* = .010), and depression (*d* = 0.40, *p* = .018). In the Intention To Treat sample, findings were attenuated, but there were still significant results on measures of rumination, self-compassion and depression.

**Conclusions:**

Findings from the present trial contribute to evidence that MBCT can lead to reduction in risk factors of depressive relapse, and strengthening of factors known to be protective of depressive relapse. The largest changes were found in the domain of self-relatedness, in the form of large effects on the participants’ ability to be less self-judgmental and more self-compassionate.

**Trial registration:**

ISRCTN, ISRCTN18001392. Registered 29 June 2018

## Background

Depression is a major public health challenge due to its prevalence and recurring nature [1–4]. Although the onset of depression is often triggered by negative life events, external circumstances seem to play a lesser role for each new depressive episode [5, 6]. Depression affects cognitive processes [7], emotional processing [8], and self-relatedness [9] in ways that heighten risk for depressive relapse. That is, those with previous experiences of depression become increasingly vulnerable to ordinary occurrences of negative thinking or difficult emotions, in that these happen more readily and are more difficult to disengage from [10]. The aim of the present study was to investigate the effects of a mindfulness-based intervention tailored to prevent depressive relapse on risk factors and protective factors within the domains of cognition, emotion and self-relatedness. In the cognitive domain, depression is associated with an increase in the intensity of rumination, a style of recurrent negative thinking in which the causes, consequences and implications of negative events and feelings are repetitively analyzed [11, 12]. It includes persistently dwelling on personal problems and inadequacies and reviewing what has gone wrong and why, in the hope that this will be of help [12]. This unproductive style of thinking is difficult to control or stop [13, 14]. The tendency to ruminate persists after depressive symptoms remit, and likely constitutes a vulnerability factor for depressive relapse [8]. Prospective studies have shown that the tendency to ruminate predicts future depressive symptoms in adults [15] and adolescents [16, 17], and may predict the onset and recurrence of depressive episodes [18, 19].

In the emotional domain, depression is characterized by an unwillingness to experience emotional reactions that may unfortunately serve to perpetuate and intensify distress [20]. A comprehensive meta-analysis found self-reported symptoms of depression to be positively associated with emotion regulation strategies trying to avoid or suppress emotions [11]. In a recent meta-analysis, individuals with recurrent depression seem to use maladaptive emotion regulation strategies and have more limited general emotion regulation abilities compared to healthy controls [8]. Another marker of depression vulnerability is emotional reactivity to stress [21]. There is a strong relationship between reactivity to stress and depression [22], and heightened emotional reactivity to daily stress has been shown to predict future depressive episodes [23, 24].

In the domain of self-relatedness, depression is characterized by self-critical attitudes and a lack of self-compassion [9, 25–27]. Remitted depressed individuals report higher levels of self-criticism and lower self-compassion than never-depressed controls [28], supporting the notion of low self-compassion as a risk factor for depressive relapse. Furthermore, levels of self-compassion has also been found to predict later levels of depressive symptoms, while levels of depressive symptoms did not predict later levels of self-compassion [29]. This indicates that self-compassion prospectively protects against depressive symptoms, rather than merely being influenced by pre-existing depression levels.

Without treatment, people suffering from recurrent depression experience relapse at rates as high as 80% [30, 31]. Based on the growing understanding of the recurring nature of Major Depressive Disorder (MDD), various treatment approaches have been developed that specifically target factors assumed to influence the risk of relapse in depression.

One such treatment approach is Mindfulness-based cognitive therapy (MBCT [32, 33]). MBCT is an eight-week group-based therapy approach that integrates selected elements of cognitive behavioral therapy for depression with the clinical application of mindfulness meditation [33]. An overarching goal of MBCT is to enable participants to relate to their own thoughts, emotions and bodily sensations with mindful awareness, and thereby break the habitual dysfunctional cycle of rumination, maladaptive emotion regulation and self-criticism that make them vulnerable for depressive relapse. There are different conceptualizations of mindfulness in the research literature, but there is general agreement that mindfulness encompasses self-regulation of attention to monitor present-moment experience, and the concurrent attitudinal qualities of acceptance, openness and non-judgment [34, 35]. In mindfulness-based interventions, participants learn an alternative to elaborating on one’s current state by way of negative repetitive thinking, or deficient coping by way of avoidance and passivity. Mindfulness could thus be a skill that lets mental and emotional states arise and pass without being prolonged by inefficient attempts to “fix” them. This skill is thought to facilitate a more “decentered” perspective, where the person is less identified with the content of thoughts and feelings and more able to view them as transient phenomena that do not need to be acted upon. And this in turn is assumed to lessen the cognitive and emotional reactivity that is seen as central to depressive relapse [36]. Mindfulness practice also emphasizes a consistent attitudinal stance of self-compassion and kindness, which may serve to counterbalance the negative self-views and judgmental thinking characteristic of depression.

There is increasing evidence that MBCT prevents depressive relapse in patients who have had multiple prior episodes of MDD [37, 38]. A number of randomised controlled trials have shown that MBCT significantly reduced the rate of relapse in recurrent major depression compared to treatment as usual [39–42]. MBCT has also proven to be as effective as long-term maintenance treatment with antidepressants in preventing relapse [43, 44]. Still, we need a better understanding of the processes by which MBCT may have its impact.

In the cognitive domain, several studies have found MBCT to reduce rumination [45–47], with effects of the intervention on depression or relapse risk either associated with or mediated by reductions in rumination [46, 48, 49]. In the emotional domain, the direct effect of MBCT on difficulties with emotion regulation and general reactivity to stress for people having recurrent depression has been less studied. One study demonstrated that MBCT participants with recurrent depression showed an overall decrease in emotional reactivity that was not present in a waitlist control group [21]. In the domain of self-relatedness, several studies have found that MBCT leads to an increase in self-compassion [31, 50]. Self-compassion is also shown to be a mediator of treatment effect [31], possibly contributing to a decoupling between depressive thinking and depressive relapse and having a key role in modulating emotional reactivity.

### Aims of the present study

MBCT has been shown to be effective both in preventing depressive relapse as well as in ameliorating ongoing depressive symptoms. Evidence also points to how MBCT reduces risk of depressive relapse, namely by altering processes of cognition (i.e. rumination) and self-relatedness (i.e. self-compassion). The effect of MBCT on risk factors within the emotional domain has however been less studied. The purpose of the present study was to further investigate the effects of MBCT on risk factors and protective factors of depressive relapse within the domains of cognition and self-relatedness as well as in the emotion domain. These processes are by no means conceptually isolated mechanisms. Rather they are interwoven and partially overlapping types of psychological functioning. For instance, scales that measure tendencies to ruminate will often seem to assess the opposite of the non-judgmental and non-reactive attitude to mental events that is found in mindfulness questionnaires. Also, measures of difficulties with emotion regulation will by necessity encompass qualities and capacities of mind that cannot easily be distinguished from mindfulness. As a final example, both mindfulness and self-compassion can be seen as capturing similar domains of experience. Nevertheless, there are separate literatures investigating the phenomena of rumination, difficulties with emotion regulation, emotional reactivity to stress, self-compassion, and mindfulness. It can be argued that these are related yet sufficiently unique constructs to warrant further investigation into how they are impacted by the MBCT intervention.

Our first hypothesis was that MBCT would be associated with a reduction of rumination, difficulties with emotion regulation, and emotional reactivity to stress. These are factors known to be a risk for depressive relapse. Although the tendency of negative repetitive thinking and difficulties with regulating emotions wanes as depressive symptoms remit, there is robust evidence that individuals with recurrent depression show a persistent tendency to ruminate and also have difficulties with emotion regulation in largely asymptomatic phases compared to people who have never been depressed [8]. Our second hypothesis was that MBCT would be associated with a strengthening of self-compassion, a factor known to protect against depressive relapse. Our third hypothesis was that MBCT would be associated with an increase in self-reported mindfulness. We assumed that the strongest findings with regard to mindfulness would be in the domains of non-judgment and non-reactivity, as these are the mindfulness facets that are most consistently cultivated in the MBCT intervention [33]. Lastly, our fourth hypothesis was that MBCT would be associated with decreased symptoms of depression and anxiety, although of a small magnitude as the included participants would be in a state of remission for depression. Nevertheless, as residual symptoms of depression is a risk factor for relapse [51], even a small reduction could be of clinical relevance.

## Method

### Setting

The data in this randomized controlled trial were collected to investigate change processes in MBCT. The study utilized a randomized wait-list controlled trial design with assessment before and after treatment. The independent variables were time (pre- and post-treatment assessments) and assigned treatment condition (MBCT or a wait-list control condition; WLC). The main dependent variables were risk and protective factors of depressive relapse: rumination, difficulties with emotion regulation, emotional reactivity to stress, and self-compassion. In addition, we registered changes in mindfulness, and symptoms of depression and anxiety. These measures allowed for detection of changes over time (pre-post) in the MBCT group while statistically controlling for within-subjects effects.

The minimum clinically meaningful difference between treatments that we wished to detect in BDI was 7. Furthermore, we accepted a probability of Type 1 error of 5% with 80% power. Kuyken et al. [43] reported an attrition rate of ca 15% in their MBCT group. We therefore expected an attrition rate to be below 20%. Given this estimate, approximately 30 participants were planned recruited to each treatment condition. A statistical power analysis (performed using G*Power) indicates that this sample size would give acceptable power.

### Participant characteristics

The participants needed to fulfill the following inclusion criteria: 18 years or older; at least three former episodes of MDD; and full or partial remission from depression. Individuals were allowed to continue use of antidepressants while participating in project, if willing to not discontinue or change their dosage during the MBCT intervention or during the wait-list period for those in the WLC condition. Individuals were excluded from participation if they fulfilled criteria of a comorbid severe mental disorder (present or life time history of psychosis, schizophrenia or bipolar disorder); fulfilled criteria for another mental disorder needing treatment, including severe obsessive compulsive disorder, post-traumatic stress disorder, severe eating disorder or borderline personality disorder; fulfilled criteria for current substance use disorders; had any neurological or hormonal diseases; had any prior or current serious cardiovascular disease; attended psychotherapy two or more times per month; had participated in a mindfulness-based intervention in the past 2 years; were pregnant or lactating.

### Sampling procedures

**Fig. 1:**
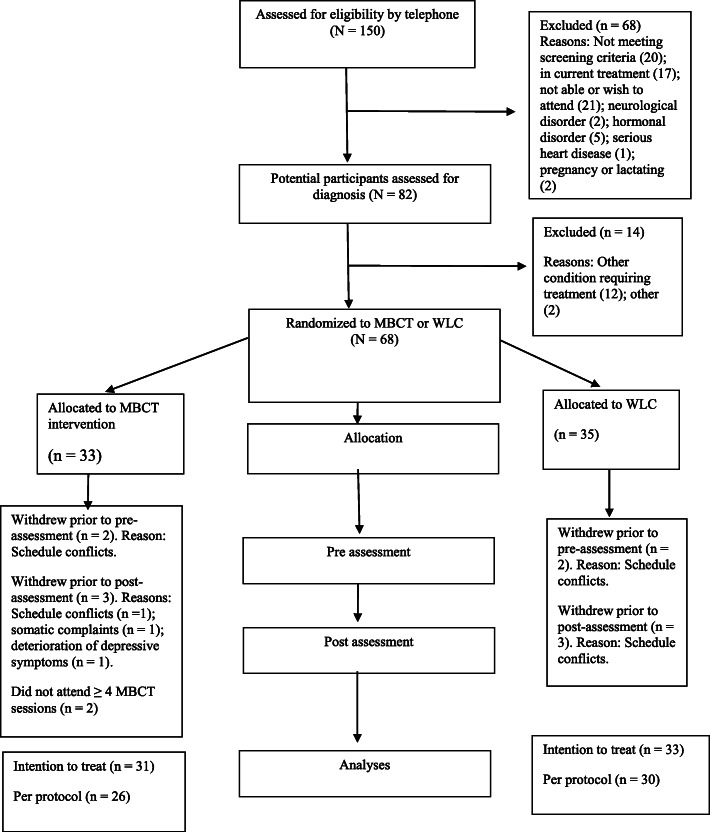
Participant flow

Participant flow is illustrated in Fig. 1. The project was registered at the ISRCTN registry (Trial no ISRCTN18001392). Participants did not receive any economic compensation, but received the prevention program free of charge. The main route for recruitment was through advertisements posted at offices and waiting rooms of general practitioners within the city of Bergen, Norway. In addition, information about the project was posted on mental health related forums on social media (Facebook). The advertisement referred to a web-page that contained additional information about the project, including a list of exclusion criteria and contact information.

After a preliminary telephone screening, 82 potential eligible participants were interviewed and assessed for eligibility. We assessed diagnostic criteria using the structured interview MINI International Neuropsychiatric Interview (MINI [52]). MINI is a structured clinical interview that assesses DSM-IV Axis I diagnoses. The interview covers 17 modules including mood and anxiety disorders, psychotic disorders, eating disorders, alcohol and substance abuse disorders and antisocial personality disorder. In addition, participants were screened for borderline personality disorder using the borderline subscale of the Structured Clinical Interview for DSM-IV (SCID-II [53]). Clinical assessments were conducted by two PhD students involved in the project (EV & JLS). The PhD students were both clinical psychologists who had undergone comprehensive training in the use of MINI, and had extensive prior experience with using the interview. The onset age of depression (assessed by retrospective recall), demographic variables, number of previous episodes of MDD and time in remission were assessed during the clinical interview. Recruitment was carried out in three separate cohorts throughout May 2016 and August 2017. The current project was an interdisciplinary collaboration featuring a wide array of outcomes, including psychophysiological measures, brain imaging data and performance-based tests of attention. Between 4 and 8 weeks after the clinical interview, all eligible participants in the study met in the laboratory at the Department of Biological and Medical psychology at the University of Bergen, Norway, where they filled out the self-report questionnaires included in the present study, as well as measures of personality traits, attachment and childhood trauma. The participants also underwent electrocardiography (ECG) measurement, and performed cognitive tests that is not reported in the present study. In addition, a sub- set of the participants in both conditions underwent an fMRI procedure. The assessors were blind to treatment condition. The total time spent in the laboratory for each participant was approximately 3 h. After completion of the pre-treatment tests, participants were randomly assigned to either the experimental- or waiting list control groups. The waiting-list period lasted for the duration of the treatment period. Participants within the WLC group did not receive any active treatment during the wait-list period, but received the MBCT program 2 weeks after post-assessment in the experimental group was completed. To ensure that the two groups matched in gender distribution, the randomization process was stratified for gender. The randomization process was carried out by a colleague not involved in the project, using the random number generation function (RAND) of Microsoft Excel (Microsoft Inc., Redmond, WA). The two PhD students involved in the project contacted the participants and assigned them to the interventions. The interventions were carried out between August 2016 and December 2017.

### Treatment

The MBCT relapse prevention intervention offered in the present study followed the standard protocol described by Segal et al. [33]. MBCT is a manualized, group-based training program designed to enable patients to learn skills that prevent the recurrence of depression. The program takes the form of eight two hour weekly group sessions, an all-day silent retreat, and individual daily homework in between sessions. The intervention was delivered in a university setting with three MBCT groups of 8–12 patients in each group. In each session, participants were introduced to a theme relevant for understanding how habitual modes of thinking, feeling, and behaving contribute to depressive relapse and to various formal and informal practices that aim to facilitate being mindful of bodily sensations, emotions and thoughts. Throughout, participants were invited to share their experiences with the practice with the instructors and other members of the group. An aim of MBCT instructors is to convey the course themes through interactive inquiry and didactic teaching, and through embodying the attitudinal components of curiosity, openness and compassion that is at the heart of the program. As the program proceeded, participants were increasingly invited to be mindful of their own adverse experiences, including thoughts, feelings and body sensations related to depression. In line with the MBCT manual [33], the participants were encouraged to practice at home between group-sessions. To aid their home practice, they were given access to recordings of formal mindfulness practices made by the instructors of their particular MBCT group. The recordings, also based on a translation of recordings used in the standard manual of Segal et al., [33] could be downloaded from a home page created for the project (https://mindfulness.w.uib.no/).

### Therapists

Three psychologists participated as instructors in the MBCT condition. All of the therapists had undergone MBCT training on a basic to intermediate level. However, none were formally certified as MBCT instructors due to limited access to training pathways nationally in Norway. All three therapists had experience of leading previous MBCT groups within the public health system in Norway, and had an ongoing personal mindfulness practice. Each of the three groups in the MBCT condition was led by a pair of the instructors (two by ES & JV and one by ES & PF).

Two complete MBCT programs were videotaped, one from each therapist pair. Three two hour sessions from each therapist pair were randomly selected. The videotapes from these sessions were translated into English and checked for therapist competency and treatment adherence by two experienced MBCT therapists independent of the trial team with the Mindfulness-Based Interventions–Teaching Assessment Criteria (MBI: TAC [54]). The MBI: TAC covers the domain of 1) coverage, pacing and organization of session curriculum, 2) relational skills, 3) embodiment of mindfulness, 4) guiding mindfulness practices, 5) conveying course themes through interactive inquiry and didactic teaching, and 6) holding the group learning environment. Each therapist is given a score per domain, a total competence/adherence score per session, and an overall score across sessions.

### Self-report measures and covariates

#### The cognitive domain

##### Rumination-reflection questionnaire (RRQ-rum [55])

The RRQ-Rum was used to assess rumination. RRQ-Rum is a 12 item questionnaire designed for measuring an individual’s stable tendency to ruminate on negative thoughts. The responses are given on a five-point Likert scale ranging from 1 (“strongly disagree”) to 5 (“strongly disagree”). Higher score on RRQ-Rum yield a greater tendency to ruminate. In the present study, we used a Norwegian translation of the RRQ-Rum which has been reported to have a high internal reliability (Cronbach’s α = .91) and to correlate negatively with self-esteem and mindfulness, and positively with habitual negative thinking [56]. RRQ-Rum showed good reliability in the current investigation [α = .87].

#### The emotional domain

##### Difficulties in emotion regulation scale (DERS [57])

The DERS was used to assess difficulties with emotion regulation. DERS is a 36 item questionnaire designed for measuring six facets of difficulties in emotion regulation. The responses are given on a five-point Likert scale ranging from 1 (almost never) to 5 (almost always). The facets include (with Cronbach’s alpha from the present study presented within brackets): (1) Lack of acceptance of emotions [α = .90]; (2) Difficulties engaging in goal-directed behavior [α = .84]; (3) Impulse control difficulties [α = .86]; (4) Lack of emotional awareness [α = .72, 5) Limited access to emotion regulation strategies [α = .83]; and (6) Lack of emotional clarity [α = .84]. Higher score on DERS yield more difficulties in emotion regulation. DERS was translated into Norwegian and validated in a Norwegian sample by Dundas, Vøllestad, Binder, and Sivertsen [58]. Recent studies have confirmed DERS’ acceptable internal consistency, construct validity and factor structure [59, 60]. The total DERS showed excellent internal consistency in the current investigation [α = .92].

##### State-trait anxiety inventory (STAI [61])

The trait-anxiety scale STAI was used to assess emotional reactivity to stress. The trait-anxiety scale of STAI, is a 20 item scale designed to measure anxiety as a personal characteristic or a trait. The responses are given on a four-point Likert scale ranging from 1 (“almost never”) to 4 (“almost always”). The trait-anxiety scale has shown excellent internal consistency (average α < .89) and test-retest reliability (average *r* = .88 [62]). We employed a Norwegian translation of the STAI [63]. The trait-anxiety scale showed good internal consistency in the current investigation (Cronbach’s α = .90).

#### The domain of self-relatedness

##### Self-compassion scale (SCS [64])

SCS was used to measure the ability to meet oneself with support and kindness when experiencing difficulties. SCS is a 26 item questionnaire designed for measuring three positive and three negative facets of how an individual treats him- or herself when experiencing difficulties in life. The responses are given on a five-point Likert scale ranging from 1 (almost never) to 5 (almost always). The positive facets include (with Cronbach’s alpha from the present study presented within brackets): (1) Self-kindness [α = .83]; (2) Common humanity [α = .79]; and (3) Mindfulness [α = .62]. The negative facets include: (1) Self-judgment [α = .82]; (2) Isolation [α = .84]; and (3) Over-identification [α = .69]. High scores on the positive subscales and low scores on the negative subscales yield a higher level of self-compassion. The SCS scale has also been used as a measure of two higher order factors [65, 66]: a strength building factor (i.e. building competency in mindfulness, common humanity, and self-kindness), and a vulnerabilities prevention factor (i.e. buffer against self-criticism, isolation, and over-identification). The positive higher order factor (composed of the positive sub-scales), and the negative higher order factor (composed of the negative sub-scales) showed identical and good internal consistency in the current investigation [α = .89]. SCS was translated into Norwegian and validated in a Norwegian sample by Dundas et al. [67]. Recent studies have confirmed that the SCS has acceptable reliability and cross-cultural validity [68]. The total SCS showed excellent internal consistency in the current investigation [α = .91].

#### Additional self-report scales

##### *Five facet mindfulness questionnaire (FFMQ* [69])

FFMQ was used to measure the ability to have a present centered awareness characterized by an attitude of openness, curiosity and acceptance. FFMQ is a 39 item questionnaire designed for measuring five facets of a trait-like general tendency to be mindful in daily life. The responses are given on a five-point Likert scale ranging from 1 (almost never true) to 5 (almost always or always true). The facets include (with Cronbach’s alpha from the present study presented within brackets): (1) Observing [α = .76]; (2) Describing [α = .94]; (3) Acting with awareness [α = .80]; (4) Non-judging [α = .88]; and (5) Non-reactivity to inner experience [α = .77]. Higher score on FFMQ yield a higher tendency to be mindful in daily life. The FFMQ has shown high construct validity [69, 70]. The five subscales are internally consistent, with alpha coefficients ranging from .76 to .91 [69]. The test-retest reliability was demonstrated to be good to excellent in a Dutch sample [71]. We employed a Norwegian translation of the FFMQ [58]. The total FFMQ showed good internal consistency in the current investigation (Chronbach’s α = .85).

##### Beck depression inventory II (BDI-II [72])

BDI-II was used to assess depressive symptoms over the past 2 weeks. BDI-II consists of 21 items that assess a range of emotional, somatic and cognitive symptoms of depression. For each item, respondents are given four options that describe the degree of a depressive symptom and are then asked to circle the most fitting option. Higher scores on BDI-II yield more severe depressive symptoms. Recent studies have confirmed BDI-II’s acceptable discriminant and convergent validity and good test–retest reliability [72]. We employed a Norwegian translation of the BDI-II [73]. BDI-II showed good internal consistency in the current investigation (Chronbach’s α = .88).

##### The Beck anxiety inventory (BAI [74])

BAI was used to assess symptoms of anxiety. BAI is a 21 item questionnaire designed for measuring the severity of anxiety symptoms. The responses are given on a three-point Likert scale ranging from 1 (never) to 3 (almost all the time). Higher scores on BAI yield more severe anxiety symptoms. Recent studies have confirmed BAI’s acceptable internal consistency [74, 75]. We employed a Norwegian translation of the BAI [76]. BAI showed good internal consistency in the current investigation (Chronbach’s α = .89).

### Ethics

The Regional Committee for Medical Research Ethics of South East Norway approved the protocols for the study (Reference: 2016/388). Before participation, written consent was obtained from all participants, and the study was conducted in accordance with the guidelines of the Declaration of Helsinki. The study adheres to the CONSORT guidelines.

### Data analysis

#### Preliminary analyses

All analyses were conducted using SPSS version 25. In preparation for the statistical analyses, self-report variables were inspected for outliers, skewness and kurtosis were inspected to ensure normal distribution, and missing values were replaced. The dataset had the following total number of missing pre and post items: Within the scale RRQ (one missing response), DERS (twenty missing responses), STAI (thirteen missing responses), SCS (twelve missing responses), FFMQ (twenty-seven missing responses), BDI (eleven missing responses), BAI (fourteen missing responses). For all variables containing missing values, comparison of means and covariances were conducted using Littles’ missing completely at random test [77]. The test indicated that all missing data were randomly distributed (all *p*s > 0.127). Missing values were therefore replaced using the expectation maximization algorithm in SPSS.

For descriptive summaries of our samples, means and standard deviations were calculated. Independent samples t-tests were conducted to test for differences between the treatment conditions in demographic variables and covariates prior to intervention. In addition, we calculated the following inference statistics: t-tests were conducted with the various outcome measures to ensure that the factors these measured were equally distributed in the treatment group and the waiting list control group. Chi-square tests were conducted with the demographic and clinical background variables. Video recordings of the MBCT treatments were evaluated and coded for adherence and competence for all three therapists involved in the MBCT condition.

#### Main analyses

One-way between-groups analyses of covariance (ANCOVA) were conducted to compare the effect of MBCT and WLC on each of the factors assumed to contribute to vulnerability for relapse in individuals with recurrent MDD following intervention, while statistically controlling for pre-intervention levels of the factor under investigation. The between-group factor was treatment condition (MBCT or WLC), and the between-group analyses were adjusted for the corresponding pre-intervention levels of each risk factor (rumination, difficulties with emotion regulation, and emotional reactivity to stress), the protective factor of self-compassion, or the additional factors of mindfulness, depression and anxiety. As the hypotheses in the present study were mainly concerned with the relative effect of completing a course of MBCT, the analyses were first performed according to the principle of completion (i.e., comprised all clients who completed both pre-and post-measurements, and attended at least four of eight MBCT sessions), in line with previous MBCT trials [41–43]. Next, identical analyses were performed on the Intention To Treat sample (ITT: i.e., all clients included in random allocation). For the subset of cases with missing post-treatment data, we used last variable carried forward to impute missing data.

## Results

None of the participants that withdrew from the study during the MBCT intervention (see Fig. 1) reported adverse effects of the MBCT intervention. Out of a total of 3 participants that withdrew, one did so due to scheduling conflicts, one experienced somatic pain that was an obstacle to partake in the mindfulness practices, and one experienced deterioration of depressive symptoms that also made it demanding to work with mindfulness practices and follow the group-based sessions. This last participant did not attribute the deterioration to the MBCT intervention.

### Preliminary analyses

#### Patient characteristics

Table 1 shows patient characteristics of the ITT sample. Independent sample t-tests were conducted on both the ITT sample and the completer sample to investigate whether the pre-levels of the various outcome measures were equally distributed in the treatment group and the waiting list control group. The results indicated that the groups did not significantly differ in total pre-scores in either of the two samples. T-test results from the ITT sample were as follows: RRQ (t (58.49) = − 0.462, *p* = .646); DERS (t (60.50) = − 0.427, *p* = .671); STAI (t (57.63) = 0.622, *p* = .511); SCS (t (61.98) = 0.781, *p* = .438); FFMQ (t (61.97) = 0.736, *p* = .464); initial levels of depression (t (60.69) = 0.408, *p* = .684); and initial level of anxiety (t (60.107) = 0.939, *p* = .352). The results of the non-significant chi-square analyses are reported in Table 1. These non-significant results imply that the randomization was successful and the participants in the two groups did not differ significantly in any of the pre-levels of demographic- or psychiatric variables, or variables used as outcome measures in the study. As no differences in baseline covariates between groups were seen, analyses were performed without adjustment for these variables.

**Table 1:** Characteristics of participants in MBCT and WLC Intention to treat sample

Variable	Total sample (*n* = 64)	MBCT (*n* = 31)	WLC (*n* = 33)	
	*Demographic characteristics*			*p*
Women: n (%)	47 (73.4)	22 (71)	25 (75.8)	0.665
Age (in years)				
M (SD)	40.1 (12.80)	40.7 (13.19)	39.5 (12.61)	0.304
Range	20–71	20–63	23–71	
Marital status n (%)				0.857
Single	21 (32.8)	11 (35.5)	10 (30.3)	
Married or cohabiting	40 (62.5)	19 (61.3)	21 (63.6)	
Separated, divorced, or widowed	3 (4.7)	1 (3.2)	2 (6.1)	
Occupational status: n (%)				0.269
Work full time	35 (54.7)	18 (58.1)	17 (51.5)	
Work part time	4 (6.3)	3 (9.7)	1 (3.0)	
Sick leave	3 (4.7)	2 (6.5)	1 (3.0)	
Unemployed/Social security	9 (14.1)	3 (9.7)	6 (18.2)	
Student	11 (17.2)	5 (16.1)	6 (18.2)	
Other	2 (3.1)		2 (6.1)	
Level of education: n (%)				0.306
High school and/or vocational qualification	15 (23.4)	9 (29)	6 (18.2)	
University degree/professional qualification	49 (76.6)	22 (71)	27 (81.8)	
	*Psychiatric characteristics*			
Depression				
HAM-D score: M (SD)	7.77 (5.38)	8.29 (5.47)	7.27 (5.34)	0.225
BDI-II score: M (SD)	12.80 (8.09)	13.23 (8.49)	12.40 (7.81)	0.579
Depression diagnosis at intake: n (%)				
In full remission	26 (40.6)	11 (35.5)	15 (45.5)	
In partial remission	19 (29.7)	11 (35.5)	8 (24.2)	
Moderate depression	19 (29.7)	9 (29)	10 (30.3)	
≥ 10 episodes: n (%)	25 (39.1)	12 (38.7)	13 (39.4)	0.955
Time (in months) since last depressive				
episode: M (SD)	17.97 (44.8)	13.81 (26.0)	21.88 (57.2)	0.351
Suicide risk: n (%)				
Low	27 (42.2)	14 (45.2)	13 (39.4)	0.641
Moderate	2 (3.1)	1 (3.2)	1 (3)	0.964
Antidepressive medication (n %)	18 (28.1)	9 (29)	9 (27.3)	0.876
Comorbidity: n (%)				
Panic disorder current	3 (4.7)	3 (9.7)	–	0.067
Agoraphobia	9 (14.1)	6 (19.4)	3 (9.1)	0.238
Social Anxiety	3 (4.7)	1 (3.2)	2 (6.1)	0.592
GAD	12 (18.8)	6 (19.4)	6 (18.2)	0.904

#### Therapist adherence and competence

On the assessment with the MBI: TAC [54], two of the therapists (ES & PF) had a proficient total competence level across sessions (level 5 of 6) and one of the therapists (JV) had an advanced total competence level across sessions (level 6 of 6). Average total adherence scale scores of 5.3 indicate acceptable adherence to protocol, and that the MBCT was delivered competently across therapists and groups.

#### Treatment effect - completer sample

An overview of changes in each of the main variables from before to after the MBCT course when participants who completed the MBCT intervention were included (completer sample), is presented in Table 2. In the cognitive domain, there was a significant medium effect of MBCT on rumination, as measured by RRQ, after controlling for the effect of pre-levels of RRQ, F (1,53) = 6.379, *p* = 0.015, *d* = 0.59.

**Table 2:** Effect of MBCT intervention on outcome – Completer sample

Variables	MBCT (*n* = 26)		WLC (*n* = 30)			
	M	SD	M	SD	F(1,53)	*ES*
RRQ						
Pre-intervention	3.9	0.6	3.9	0.5		
Post-intervention	3.5	0.6	3.8	0.4	6.379*	0.59
DERS						
Pre-intervention	105.5	17.9	106.6	23.8		
Post-intervention	95.6	23.5	107.6	24.5	5.076*	0.50
STAI-trait						
Pre-intervention	52.8	9.6	51.3	7.4		
Post-intervention	46.3	10.5	49.6	9.9	4.097*	0.32
SCS						
Pre-intervention	15.1	3.1	14.7	3.7		
Post-intervention	18.5	4.9	14.1	3.6	26.704***	1.02
FFMQ						
Pre-intervention	14.6	1.8	14.3	2.0		
Post-intervention	15.7	2.6	14.3	2.1	7.155**	0.59
BDI-II						
Pre-intervention	14.6	8.3	12.8	8.0		
Post-intervention	10.8	9.5	14.5	9.1	5.919*	0.40
BAI						
Pre-intervention	14.8	6.8	12.7	9.0		
Post-intervention	13.1	6.7	13.8	10.5	2.766	–

Abbreviations: *MBCT* Mindfulness Based Cognitive Therapy, *WLC* Waiting-list Control, *M* mean, *SD* standard deviation, *ES* effect size (Cohen’s d), *RRQ* Rumination-Reflection Questionnaire, *DERS* Difficulties in Emotion Regulation Scale (total score), *STAI-trait* trait–anxiety scale of the State-Trait Anxiety Inventory, *SCS* Self-compassion Scale (total score), *FFMQ* Five Facet Mindfulness Questionnaire (total score), *BDI-II* Beck Depression Inventory, *BAI* Beck Anxiety Inventory. ∗*p* < 0.05; ∗∗*p* < 0.01; ∗∗∗*p* < 0.001

In the emotional domain, there was a significant medium effect of MBCT on difficulties in emotion regulation, measured with DERS, after controlling for the effect of pre-levels of DERS, F (1,53) = 5.076, *p* = 0.028, *d* = 0.50. An overview of changes in each subscale of the DERS from before to after the MBCT course is presented in Table 3.

**Table 3:** ANCOVA analyses showing changes and effect sizes in subscales of DERS, SCS, and FFMQ when comparing post values of MBCT and WLC statistically controlled for pre-intervention levels of the subscale under investigation

Variables	MBCT (*n* = 26)		WLC (*n* = 30)			
	M	SD	M	SD	F(1,53)	*ES*
**DERS**						
Non-acceptance	15.5	7.0	19.7	4.9	6.062*	0.69
Strategies	20.5	6.8	24.5	7.4	5.968*	0.56
Clarity	11.7	4.3	13.4	4.5	4.267*	0.39
Goals	16.3	3.4	17.0	4.7	2.157	–
Impulse	13.9	4.2	14.9	6.2	0.731	–
Awareness	16.5	4.0	17.0	4.7	0.190	–
**SCS**						
Self-judgment	3.5	1.0	2.5	0.8	24.146***	1.10
Over-identification	3.3	0.7	2.5	0.8	24.208***	1.06
Self-kindness	2.8	1.0	2.1	0.6	14.532***	0.85
Mindfulness	2.8	0.9	2.2	0.6	16.630***	0.78
Common humanity	2.9	1.0	2.2	0.8	11.250***	0.77
Isolation	3.2	1.1	2.8	1.0	3.419	–
**FFMQ**						
Non-judgment	3.3	0.8	2.6	0.7	7.509**	0.93
Non-reactivity	3.0	0.7	2.6	0.5	6.33*	0.66
Observe	3.5	0.6	3.2	0.7	4.551*	0.46
Describe	3.1	1.0	3.1	0.8	2.308	–
Act with awareness	2.8	0.6	2.7	0.7	0.227	–

Abbreviations: *MBCT* Mindfulness Based Cognitive Therapy, *WLC* Waiting-list Control, *M* mean, *SD* standard deviation, *ES* effect size (Cohen’s d), *DERS* Difficulties in Emotion Regulation Scale, *SCS* Self-compassion Scale, *FFMQ* Five Facet Mindfulness Questionnaire; Scores on DERS, SCS and FFMQ ranges from 1 (Almost never) to 5 (Almost always). ∗*p* < 0.05; ∗∗*p* < 0.01; ∗∗∗*p* < 0.001

There was also a significant small effect of mindfulness training on emotional reactivity to stress, as measured by STAI-trait, after controlling for the effect of pre-levels of STAI trait, F (1,53) = 4.097, *p* = 0.048, *d* = 0.32.

In the domain of self-relatedness, there was a large and significant effect of mindfulness training on increased levels of self-compassion, as measured by SCS, after controlling for the effect of pre-levels of SCS, F (1,53) = 26.704, *p* < 0.001, *d* = 1.02. There was a large and significant effect of mindfulness training on a strength building modality, measured by the positive sub-scales of SCS, after controlling for the pre-levels of the positive sub-scales, F (1,53) = 17.683, *p* < 0.001, *d* = 0.91. There was also a large and significant effect of mindfulness training on a vulnerabilities prevention modality, measured by the negative subscales of SCS, F (1,53) = 19.353, *p* < 0.001, *d* = 0.90. An overview of changes in each subscale of the SCS from before to after the MBCT course is presented in Table 3.

Furthermore, there was a significant medium effect of MBCT on total mindfulness, as measured by FFMQ, after controlling for the effect of pre-levels of FFMQ, F (1,53) = 7.155, *p* = 0.010, *d* = 0.59. An overview of changes in each subscale of the FFMQ from before to after the MBCT course is presented in Table 3.

Lastly, there was a significant small effect of MBCT on symptoms of depression, as measured with BDI, after controlling for the effect of pre-levels of BDI, F (1,53) = 5.92, *p* = 0.018, *d* = 0.40. There was not a significant effect of MBCT on symptoms of anxiety, as measured by BAI, after controlling for the effect of pre-levels of BAI, F (1,53) = 2.766, *p* = 0.102.

#### Treatment effect – intention to treat sample

An overview of changes in each of the main variables from before to after the MBCT course when all participants randomized into the study were included (Intention to treat sample), is presented in Table 4. Treatment effects on measures of rumination (RRQ), self-compassion (SCS), and depression (BDI-II) were still significant in the ITT sample (see Table 4). Within the SCS data, both the strength building modality (F (1,61) = 12.63, *p* = 0.001, *d* = 0.85) and the vulnerabilities prevention modality of the SCS (F (1,61) = 11.60, *p* = 0.001, *d* = 0.66) were also significant in the ITT sample.

**Table 4:** Effect of MBCT intervention on outcome – Intention to Treat sample

Variables	MBCT (*n* = 31)		WLC (*n* = 33)			
	M	SD	M	SD	F(1,53)	*ES*
RRQ						
Pre-intervention	3.8	0.7	3.9	0.5		
Post-intervention	3.5	0.7	3.7	0.4	4.595*	0.40
DERS						
Pre-intervention	102.1	19.1	104.4	23.8		
Post-intervention	96.2	24.3	105.3	24.6	2.259	–
STAI-trait						
Pre-intervention	51.7	10	50.2	8.0		
Post-intervention	47.2	10.1	48.7	10	1.135	–
SCS						
Pre-intervention	15.8	3.5	15.1	3.8		
Post-intervention	18.3	4.7	14.5	3.8	15.823***	0.89
FFMQ						
Pre-intervention	14.9	1.9	14.5	2.0		
Post-intervention	15.5	2.5	14.5	2.1	2.526	–
BDI-II						
Pre-intervention	13.2	8.5	12.4	7.8		
Post-intervention	10.4	9.1	13.9	8.9	5.120*	0.39
BAI						
Pre-intervention	14.2	6.8	12.4	8.7		
Post-intervention	13.1	8.2	13.4	10.2	1.455	–

Abbreviations: *MBCT* Mindfulness Based Cognitive Therapy, *WLC* Waiting-list Control, *M* mean, *SD* standard deviation, *ES* effect size (Cohen’s d), *RRQ* Rumination-Reflection Questionnaire, *DERS* Difficulties in Emotion Regulation Scale (total score), *STAI-trait* trait–anxiety scale of the State-Trait Anxiety Inventory, *SCS* Self-compassion Scale (total score), *FFMQ* Five Facet Mindfulness Questionnaire (total score), *BDI-II* Beck Depression Inventory, *BAI* Beck Anxiety Inventory. ∗*p* < 0.05; ∗∗*p* < 0.01; ∗∗∗*p* < 0.001

Six participants from the ITT sample did not complete treatment and post-measurements (3 in the MBCT condition and 3 in the WCL condition), and two participants in the MCBT condition did not attend a minimum of 4 MBCT sessions. Seven of these eight participants reported to have experienced more than 10 previous episodes of depression.

## Discussion

Major Depressive Disorder (MDD) can develop into a chronic disorder that constitutes a significant public health challenge. Mindfulness-Based Cognitive Therapy (MBCT) is one of several interventions aiming to reduce the risk of recurrent depressive episodes. The aim of the current study was to investigate the effects of MBCT on particular risk and protective factors of depressive relapse within the cognitive domain, the emotional domain and the domain of self-relatedness. These domains are explicit targets of MBCT, and the processes of rumination, emotion regulation and self-compassion have been found in recent reviews and meta-analyses to be likely mechanisms of change in mindfulness-based interventions [78–80]. Our findings show that completing an MBCT course led to significant changes in the expected direction in all three domains.

In line with our first hypothesis, we found that MBCT was associated with a reduction of rumination, difficulties with emotion regulation, and emotional reactivity to stress. In the cognitive domain, MBCT participants experienced a medium sized decrease in ruminative thinking, measured with the Rumination-Reflection Questionnaire (RRQ). This suggests that mindfulness training did help the participants to control the tendency to focus attention on repetitive thoughts concerning the reasons and consequences for negative feelings. This supports previous findings that MBCT is an intervention that aids participants rehearsed in rumination to control this unproductive style of thinking and vulnerability factor for depressive relapse [49]. The finding is also in line with theoretical assumptions in MBCT that practicing becoming aware of thoughts and relating to them non-judgmentally, or learning to shift attention from thoughts to the felt sense of experience in the present moment, helps participants disengage from negative repetitive thinking [42].

In the emotional domain, MBCT participants experienced a small to medium effect on emotion regulation, measured with the Difficulties in Emotion-Regulation Scale (DERS). The most prominent finding was on the subscale of *non-acceptance*. This captures a tendency to allow difficult emotions to come and go without trying to suppress or change them. Relatedly, the subscale of *strategies* encompasses beliefs that difficult emotions will pass and a sense of self-efficacy with regard to dealing with them. Both of these effects were of a moderate magnitude, indicating that MBCT participants experienced changes in their ability to manage emotions in line with what is taught in the intervention. Mindful emotion regulation involves the non-judgmental awareness of affective states as transient phenomena. Attending to aversive experiences with an attitude of acceptance, instead of trying to decrease or remove these experiences, is assumed to increase tolerance for difficult emotions and lessen emotional reactivity. This stance of noticing and letting be is in contrast to the maladaptive emotion regulatory processes found to be associated with depression, such as suppression and avoidance [11, 81–83]. The effect on the subscale *clarity* was small but significant– indicating that there was somewhat of a tendency in participants to become more aware of and able to discern and understand emotional reactions.

Also, in the emotional domain, MBCT participants experienced a small positive impact on their tendency to attend to, experience and report negative emotions, such as fear, worry and anxiety across situations, measured with the State-Trait Anxiety Inventory (STAI). This could be indicative of MBCT contributing to emotional stability. The results are in line with findings from Britton et al. [21], with MBCT participants showing an overall decrease in emotional reactivity that was not present in the waitlist control group. Furthermore, the changes in emotional reactivity partially mediated improvements in symptoms of depression. The results are also in line with findings from Sharplin, Jones, Hancock, Knott, Bowden, & Whitford [84], investigating the effect of MBCT for patients with cancer who also experienced symptoms of depression and anxiety.

In line with our second hypothesis, MBCT was associated with a strengthening of self-compassion, as measured by the Self-Compassion Scale (SCS). It is notable that the strongest findings in the current study were within the domain of self-relatedness, in the form of the participants’ increased ability to be accepting and supportive towards themselves in times of suffering. The large effect of MBCT on self-compassion indicates that what is being cultivated during MBCT is not mere equanimity or a form of adaptive disengagement from mental and emotional content. Rather, our findings show that participants are able to relate to experience with a greater sense of warmth, kindness and benevolence. The strong effect of MBCT on participants’ self-compassion is in line with Kuyken et al. [43], who found that self-compassion seemed to reduce cognitive reactivity and mediate the effect of MBCT treatment on depressive relapse, and concluded that self-compassion is a key skill learned in MBCT.

The active countering of self-criticism with a compassionate perspective may be particularly important, as depression is characterized by severe and categorical negative judgments about oneself, others and the world. In the present study, we also found a consistent pattern with five of six subscales showing significant effects, three of which are of large magnitude and two medium to large. Participants clearly indicate that they have become less judgmental and kinder toward themselves. Furthermore, the scores on the subscale of *over-identification* points to changes in the relationship to one’s own emotional state – from a proneness to being overly absorbed and overwhelmed, to gaining a healthy sense of perspective. The subscale of *common humanity* captures a dimension unique to the self-compassion construct, namely the tendency to view ongoing difficulties as a universal feature of life instead of being due to personal weakness and insufficiencies. The robust changes on this subscale in the current study could thus indicate that participants have become more accepting and less judgmental about their own vulnerabilities – a stance very much in keeping with the curriculum of MBCT concerning depression.

The only null finding with regard to self-compassion was for the *isolation* subscale. This subscale assesses a tendency to lose a sense of belonging when experiencing difficulties, with concomitant self-criticism. We find this somewhat puzzling, as the positive effects on *common humanity* were so large. It is possible that the items of this subscale taps a type of negatively charged self-relatedness that is more deep-seated and thus would require longer time to change. Another possibility is that feeling less isolated does not necessarily mean one feels more connected to the rest of humanity. Lopez and colleagues [65] have argued that the best use of the SCS scale is to measure two separate factors. A strength building factor consisting of the positive subscales (i.e. building competency in mindfulness, common humanity, and self-kindness), and a vulnerability prevention factor, consisting of the positive subscales (i.e. buffer against self-criticism, isolation, and over-identification). In line with this argument, the isolation subscale is best understood as part of a vulnerability factor, and not as the converse of common humanity [65]. In the present study, MBCT was found as a method to strengthen both these factors.

In line with our third hypothesis, we also found a medium sized increase in self-reported mindfulness in the form of significant effects on the total score of the Five Facet Mindfulness Questionnaire (FFMQ). This effect was largely driven by large size increases in the facets of *non-judgment* and a medium sized increase in *non-reactivity*, validating that MBCT had a positive impact on the participants’ ability to accept their own thoughts and emotions and be less prone to react to them in habitual and maladaptive ways. This is important, as mindfulness is the central capacity that is being cultivated in MBCT. It is also the case that an open and accepting attitude toward one’s own experiences is assumed to be of particular relevance in alleviating depression and other internalizing disorders. Previous trials of MBCT have found changes in mindfulness during treatment to be associated with reduced risk for relapse [31, 85]. *Non-judgement* has also been found to predict depressive symptoms 2 years later [86]. The theoretical assumption is that a stance of equanimity enables distress to fluctuate naturally, without the individual contributing to further distress by maladaptive emotion regulation strategies. In this regard, findings from both the DERS and FFMQ indicate that MBCT participants have become more skilled at managing difficult experiences without becoming absorbed in them or trying to suppress or escape them. The reported reduction in the tendency to ruminate further supports the emergence of a more allowing stance with regard to negative and repetitive thinking.

Conversely, the null findings for the other facets of mindfulness could point to these being less crucial to the clinical application of mindfulness. The subscale of *observe* did show change, albeit with a small effect size. This facet of mindfulness has been debated, as there are contradictory findings both with regard to its relationship to the overall factor of mindfulness [69] and its relationship to mental health and well-being [86]. There is an emerging consensus that the ability to observe one’s own experience can be both adaptive and maladaptive, depending on the presence of concurrent capacities for non-judgment and non-reactivity [87]. In the absence of these attitudinal aspects of mindfulness, an elevated observe scale could indicate aversive forms of self-awareness. However, in the present sample, the notable increase in non-judgment and non-reactivity lends support to participants having learned a more beneficial mode of self-observation during MBCT.

Finally, in line with our forth hypothesis, there was also a reduction of depressive symptoms as measured by the Beck Depression Inventory (BDI-II). There are clear limitations as to how large an impact we could expect to find, as it was a prerequisite for inclusion that participants were not acutely depressed. Nevertheless, the small to medium sized reduction in BDI scores is relevant as research indicates that residual depressive symptoms increase the risk of relapse [88]. However, we did not find as hypothesized that MBCT was associated with a significant reduction in anxiety as measured with the Beck Anxiety Inventory (BAI).

When performing the more conservative analyses including the patients who did not attend MBCT, or attended less than a minimum of 4 sessions, we found that only significant changes in rumination, self-compassion and depression remained. Nevertheless, these can be regarded as key outcomes in the preventing of depressive relapse [31]. The results from the ITT analyzes confirmed the main conclusion of this manuscript, that is that the largest effect of MBCT seems to be in the domain of self-relatedness, in the form of large effects on the participants’ ability to be less self-judgmental and more self-compassionate.

One purpose of an ITT analysis is to investigate whether there are systematic differences between treatment completers and non-completers. In the present study, seven out of eight patients who dropped out of either the MBCT or the WCL condition reported having experienced more than ten previous episodes of depression. This could indicate that a more chronic course of recurrent depression can make it more challenging to complete MBCT. However, this needs to be confirmed in other studies to investigate that this is a systematic tendency in MBCT, and not a random finding in the present study. One possible implication of this finding might be to take extra care preparing participants with a high number of previous depressive episodes that they might lose hope or motivation, and offer them the possibility to get in touch and discuss their decision before giving up on treatment.

### Strengths and limitations

A strength of the present study was that independent researchers completed the recruitment, diagnostics and assessments of the participants. Still, participants may have been influenced by factors such as social desirability, wanting to be perceived as socially acceptable rather than according to how he or she truly behaved or felt. It is nevertheless of interest that in the current study, there is a differential pattern of effects in that not all subscales on questionnaires show significant changes.

Another strength of the study is that treatment integrity was assessed by independent raters, and all therapists involved in the study showed satisfying levels of competence and adherence.

One main limitation of this study was the absence of an active control group, which means we cannot exclude the possible impact of non-specific factors, such as being part of a group, receiving care and attention or other group related factors. This means that the improvement experienced by the MBCT participants may in part be due to their expectations to improve, that is, placebo effect. However, previous studies, which did include an active control, have demonstrated the effect of MBCT for individuals with recurrent depression [89–91].

Another limitation is that the study is solely based on self-report measures. Answering questions related to one’s own mental habits and behaviors is a difficult cognitive task [92], and it is likely that participants do not have a detailed memory or insight into all relevant behavior. Participants’ self-ratings might represent their own perceptions of levels of self-compassion and mindfulness rather than “objective” or true levels, and the validity thus relies on participants’ memory, honesty and introspective abilities (e.g. [93]).

Using the BDI-II as an outcome variable to calculate sample size may not have been the most optimal choice. As most of the participants were between depressive episodes and had a relatively low level of depressive symptoms at pre-treatment, it would have been more suitable to estimate power based on a measure that one would expect to be more impacted by the intervention, such as level of rumination/self-criticism. However, post-hoc power analyses using G*Power3, [94] revealed an observed sufficient power (above .80) on the main measures of rumination and self-compassion in the completer sample.

The assessors in the present study were blind to treatment condition. However, at post-treatment, some of the participants did comment on the effect of the MBCT course. It was therefore difficult to uphold a totally uninformed perspective.

The participants in the present study were relatively homogenous, especially when it comes to education. As a group, the participants had a quite high level of education, which may limit the generalizability of the findings.

### Future research

As a direct continuation of the present study, it would of relevance to investigate to what degree the various vulnerability and protective factors are associated with reduced occurrence of depressive relapse after 6, 12 and 24 months. Also, it would be of interest to investigate whether changes in vulnerability and protective factors of depressive relapse are associated with performance on cognitive tests, measures of heart rate variability and functional MRI.

## Conclusion

MBCT is tailored to prevent depressive relapse. The present study validates MBCT as an intervention that has an effect on risk factors and protective factors of depressive relapse within the cognitive domain, emotional domain and the domain of self-relatedness. The main change was within the domain of self-relatedness, in the form of positive changes in participants’ level of self-judgement and self-compassion.

## Data Availability

This dataset are available by contacting the corresponding author ES. The trial protocol can be made available by contacting the corresponding author ES.

## Abbreviations

MBCT: Mindfulness based cognitive therapy
WLC: Wait-list control
MDD: Major depressive disorder
RRQ: Rumination-reflection questionnaire
DERS: Difficulties with emotion regulation scale
STAI: State-trait anxiety inventory
SCS: Self-compassion scale
FFMQ: Five facet mindfulness questionnaire
BDI: Beck’s depression inventory
BAI: Beck’s anxiety inventory

## References

[1] Barnhofer T, Crane C, Hargus E, Amarasinghe M, Winder R, Williams JMG (2009). Mindfulness-based cognitive therapy as a treatment for chronic depression: a preliminary study. Behav Res Ther.

[2] Kessler RC, Bromet EJ (2013). The epidemiology of depression across cultures. Annu Rev Public Health.

[3] Williams JMG (2008). Mindfulness, depression and modes of mind. Cogn Ther Res.

[4] World Health Organization (2017). Depression and other common mental disorders.

[5] Kessing LV, Andersen PK (2004). Does the risk of developing dementia increase with the number of episodes in patients with depressive disorder and in patients with bipolar disorder?. J Neurol Neurosurg Psychiatry.

[6] Monroe SM, Harkness KL (2005). Life stress, the" kindling" hypothesis, and the recurrence of depression: considerations from a life stress perspective. Psychol Rev.

[7] Kircanski K, Joormann J, Gotlib IH (2012). Cognitive aspects of depression. Wiley Interdiscip Rev Cogn Sci.

[8] Visted E, Vøllestad J, Nielsen MB, Schanche E (2018). Emotion regulation in current and remitted depression: a systematic review and meta-analysis. Front Psychol.

[9] MacBeth A, Gumley A (2012). Exploring compassion: a meta-analysis of the association between self-compassion and psychopathology. Clin Psychol Rev.

[10] Segal ZV, Kennedy S, Gemar M, Hood K, Pedersen R, Buis T (2006). Cognitive reactivity to sad mood provocation and the prediction of depressive relapse. Arch Gen Psychiatry.

[11] Aldao A, Nolen-Hoeksema S, Schweizer S (2010). Emotion regulation strategies across psychopathology: a metaanalytic review. Clin Psychol Rev.

[12] Baer RA (2007). Mindfulness, assessment, and transdiagnostic processes. Psychol Inq.

[13] Joormann J, Vanderlind WM (2014). Emotion regulation in depression: the role of biased cognition and reduced cognitive control. Clin Psychol Sci.

[14] Nolen-Hoeksema S, Wisco BE, Lyubomirsky S (2008). Rethinking rumination. Perspect Psychol Sci.

[15] Nolen-Hoeksema S, Parker LE, Larson J (1994). Ruminative coping with depressed mood following loss. J Pers Soc Psychol.

[16] Broderick PC, Korteland C (2004). A prospective study of rumination and depression in early adolescence. Clinical Child Psychology and Psychiatry.

[17] Nolen-Hoeksema S, Stice E, Wade E, Bohon C (2007). Reciprocal relations between rumination and bulimic, substance abuse, and depressive symptoms in female adolescents. J Abnorm Psychol.

[18] Nolen-Hoeksema S (1991). Responses to depression and their effects on the duration of depressive episodes. J Abnorm Psychol.

[19] Robinson MS, Alloy LB (2003). Negative cognitive styles and stress-reactive rumination interact to predict depression: a prospective study. Cogn Ther Res.

[20] Yoon S, Dang V, Mertz J, Rottenberg J (2018). Are attitudes towards emotions associated with depression? A conceptual and meta-analytic review. J Affect Disord.

[21] Britton WB, Shahar B, Szepsenwol O, Jacobs WJ (2012). Mindfulness-based cognitive therapy improves emotional reactivity to social stress: results from a randomized controlled trial. Behav Ther.

[22] Endler NS, Parker JD (1990). State and trait anxiety, depression and coping styles. Aust J Psychol.

[23] Cohen LH, Gunthert KC, Butler AC, O'Neill SC, Tolpin LH (2005). Daily affective reactivity as a prospective predictor of depressive symptoms. J Pers.

[24] Pine DS, Cohen P, Brook J (2001). Adolescent fears as predictors of depression. Biol Psychiatry.

[25] Gilbert P, Baldwin MW, Irons C, Baccus JR, Palmer M (2006). Self-criticism and self-warmth: an imagery study exploring their relation to depression. J Cogn Psychother.

[26] Krieger T, Altenstein D, Baettig I, Doerig N, Holtforth MG (2013). Self-compassion in depression: associations with depressive symptoms, rumination, and avoidance in depressed outpatients. Behav Ther.

[27] Luyten P, Sabbe B, Blatt SJ, Meganck S, Jansen B, De Grave C (2007). Dependency and self-criticism: relationship with major depressive disorder, severity of depression, and clinical presentation. Depression Anxiety.

[28] Ehret AM, Joormann J, Berking M (2015). Examining risk and resilience factors for depression: the role of self-criticism and self-compassion. Cognit Emot.

[29] Krieger T, Berger T, Holtforth M (2016). The relationship of self-compassion and depression: cross-lagged panel analyses in depressed patients after outpatient therapy. J Affect Disord.

[30] Kupfer DJ, Frank E, Perel JM, Cornes C, Mallinger AG, Thase ME (1992). Five-year outcome for maintenance therapies in recurrent depression. Arch Gen Psychiatry.

[31] Kuyken W, Watkins E, Holden E, White K, Taylor RS, Evans A (2010). How does mindfulness-based cognitive therapy work?. Behav Res Ther.

[32] Segal ZV, Williams JMG, Teasdale JD (2002). Mindfulness-based cognitive therapy for depression.

[33] Segal ZV, Williams JMG, Teasdale JD (2013). Mindfulness-based cognitive therapy for depression.

[34] Bishop SR, Lau M, Shapiro S, Carlson L, Anderson ND, Carmody J (2004). Mindfulness: a proposed operational definition. Clin Psychol Sci Pract.

[35] Creswell JD, Lindsay EK, Villalba DK, Chin B (2019). Mindfulness training and physical health: mechanisms and outcomes. Psychosom Med.

[36] Bylsma LM, Morris BH, Rottenberg J (2008). A meta-analysis of emotional reactivity in major depressive disorder. Clin Psychol Rev.

[37] Kuyken W, Warren FC, Taylor RS, Whalley B, Crane C, Bondolfi G (2016). Efficacy of mindfulness-based cognitive therapy in prevention of depressive relapse: an individual patient data meta-analysis from randomized trials. JAMA Psychiat.

[38] MacKenzie MB, Abbott KA, Kocovski NL (2018). Mindfulness-based cognitive therapy in patients with depression: current perspectives. Neuropsychiatr Dis Treat.

[39] Bondolfi G, Jermann F, Van der Linden M, Gex-Fabry M, Bizzini L, Rouget BW (2010). Depression relapse prophylaxis with mindfulness-based cognitive therapy: replication and extension in the Swiss health care system. J Affect Disord.

[40] Godfrin KA, Van Heeringen C (2010). The effects of mindfulness-based cognitive therapy on recurrence of depressive episodes, mental health and quality of life: a randomized controlled study. Behav Res Ther.

[41] Ma SH, Teasdale JD (2004). Mindfulness-based cognitive therapy for depression: replication and exploration of differential relapse prevention effects. J Consult Clin Psychol.

[42] Teasdale JD, Segal ZV, Williams JMG, Ridgeway VA, Soulsby JM, Lau MA (2000). Prevention of relapse/recurrence in major depression by mindfulness-based cognitive therapy. J Consult Clin Psychol.

[43] Kuyken W, Byford S, Taylor RS, Watkins E, Holden E, White K (2008). Mindfulness-based cognitive therapy to prevent relapse in recurrent depression. J Consult Clin Psychol.

[44] Segal ZV, Bieling P, Young T, MacQueen G, Cooke R, Martin L (2010). Antidepressant monotherapy vs sequential pharmacotherapy and mindfulness-based cognitive therapy, or placebo, for relapse prophylaxis in recurrent depression. Arch Gen Psychiatry.

[45] Keune PM, Bostanov V, Hautzinger M, Kotchoubey B (2011). Mindfulness-based cognitive therapy (MBCT), cognitive style, and the temporal dynamics of frontal EEG alpha asymmetry in recurrently depressed patients. Biol Psychol.

[46] Michalak J, Hölz A, Teismann T (2011). Rumination as a predictor of relapse in mindfulness-based cognitive therapy for depression. Psychol Psychother Theory Res Pract.

[47] van den Hurk PAM, van Aalderen JR, Giommia F, Donders RART, Barendregt HP, Speckens AEM (2012). An investigation of the role of attention in mindfulnessbased cognitive therapy for recurrently depressed patients. J Experiential Psychopathol.

[48] Shahar B, Britton WB, Sbarra DA, Figueredo AJ, Bootzin RR (2010). Mechanisms of change in mindfulness-based cognitive therapy for depression: preliminary evidence from a randomized controlled trial. Int J Cogn Ther.

[49] van Aalderen JR, Donders ART, Giommi F, Spinhoven P, Barendregt HP, Speckens AEM (2012). The efficacy of mindfulness-based cognitive therapy in recurrent depressed patients with and without a current depressive episode: a randomized controlled trial. Psychol Med.

[50] Rimes KA, Wingrove J (2013). Mindfulness-based cognitive therapy for people with chronic fatigue syndrome still experiencing excessive fatigue after cognitive behavior therapy: a pilot randomized study. Clinical psychology & psychotherapy.

[51] Paykel ES (2008). Partial remission, residual symptoms, and relapse in depression. Dialogues Clin Neurosci.

[52] Sheehan DV, Lecrubier Y, Sheehan KH, Amorim P, Janavs J, Weiller E (1998). The MINI-international neuropsychiatric interview (MINI): the development and validation of a structured diagnostic psychiatric interview for DSM-IV and ICD-10. J Clin Psychiatry.

[53] Gibbon M, Spitzer RL, Williams JBW, Benjamin LS, First MB (1997). Structured clinical interview for DSM-VI axis II personality disorders (SCID-II): American Psychiatric Publishing.

[54] Crane RS, Eames C, Kuyken W, Hastings RP, Williams JMG, Bartley T (2013). Development and validation of the mindfulness-based interventions–teaching assessment criteria (MBI: TAC). Assessment.

[55] Trapnell PD, Campbell JD (1999). Private self-consciousness and the five-factor model of personality: distinguishing rumination from reflection. J Pers Soc Psychol.

[56] Verplanken B, Friborg O, Wang CE, Trafimow D, Woolf K (2007). Mental habits: metacognitive reflection on negative self-thinking. J Pers Soc Psychol.

[57] Gratz KL, Roemer L (2004). Multidimensional assessment of emotion regulation and dysregulation: development, factor structure, and initial validation of the difficulties in emotion regulation scale. J Psychopathol Behav Assess.

[58] Dundas I, Vøllestad J, Binder PE, Sivertsen B (2013). The five factor mindfulness questionnaire in Norway. Scand J Psychol.

[59] Fowler JC, Charak R, Elhai JD, Allen JG, Frueh BC, Oldham JM (2014). Construct validity and factor structure of the difficulties in emotion regulation scale among adults with severe mental illness. J Psychiatr Res.

[60] Ritschel LA, Tone EB, Schoemann AM, Lim NE (2015). Psychometric properties of the difficulties in emotion regulation scale across demographic groups. Psychol Assess.

[61] Spielberger CD (1983). Manual for the state-trait anxiety inventory STAI (form Y)(“ self-evaluation questionnaire”).

[62] Barnes LL, Harp D, Jung WS (2002). Reliability generalization of scores on the Spielberger state-trait anxiety inventory. Educ Psychol Meas.

[63] Haseth K, Hagtvet K, Spielberger CD, Spielberger CD, Diaz-Guerrero R, Strelau J (1990). Psychometric properties and research with the norwegian state-trait anxiety inventory. In cross-cultural anxiety.

[64] Neff KD (2003). The development and validation of a scale to measure self-compassion. Self Identity.

[65] López A, Sanderman R, Smink A, Zhang Y, Van Sonderen E, Ranchor A, Schroevers MJ. A reconsideration of the self-compassion Scale’s total score: self-compassion versus self-criticism. PLoS One. 2015;10(7). 10.1371/journal.pone.0132940. 10.1371/journal.pone.0132940 PMC4508060 26193654

[66] Wan HYA, [溫浩然] (2018). Self-compassion and bio-psychosocial well-being: the application of mindful self-compassion training on cancer survivors in Hong Kong.

[67] Dundas I, Svendsen JL, Wiker AS, Granli KV, Schanche E (2016). Self compassion and depressive symptoms in a Norwegian student sample. Nordic Psychology.

[68] Neff KD, Pisitsungkagarn K, Hsieh YP (2008). Self-compassion and self-construal in the United States, Thailand, and Taiwan. J Cross-Cult Psychol.

[69] Baer RA, Smith GT, Hopkins J, Krietemeyer J, Toney L (2006). Using self-report assessment methods to explore facets of mindfulness. Assessment.

[70] Baer RA, Smith GT, Lykins E, Button D, Krietemeyer J, Sauer S (2008). Construct validity of the five facet mindfulness questionnaire in meditating and nonmeditating samples. Assessment.

[71] Bohlmeijer E, Ten Klooster PM, Fledderus M, Veehof M, Baer R (2011). Psychometric properties of the five facet mindfulness questionnaire in depressed adults and development of a short form. Assessment.

[72] Beck AT, Steer RA, Brown GK (1996). Beck depression inventory-II (BDI-II) manual.

[73] Aasen H (2001). An empirical investigation of depression symptoms: norms, psychometric characteristics and factor structure of the Beck depression inventory II.

[74] Beck AT, Steer RA (1988). Beck anxiety inventory (BAI). Überblick über Reliabilitäts-und Validitätsbefunde von klinischen und außerklinischen Selbst-und Fremdbeurteilungsverfahren.

[75] Fydrich T, Dowdall D, Chambless DL (1992). Reliability and validity of the Beck anxiety inventory. Journal of anxiety disorders.

[76] Nordhagen T (2001). Beck Anxiety Inventory: translation and validation of a Norwegian version.

[77] Little RJ (1988). A test of missing completely at random for multivariate data with missing values. J Am Stat Assoc.

[78] Alsubaie M, Abbott R, Dunn B, Dickens C, Keil TF, Henley W (2017). Mechanisms of action in mindfulness-based cognitive therapy (MBCT) and mindfulness-based stress reduction (MBSR) in people with physical and/or psychological conditions: a systematic review. Clin Psychol Rev..

[79] Gu J, Strauss C, Bond R, Cavanagh K (2015). How do mindfulness-based cognitive therapy and mindfulness-based stress reduction improve mental health and wellbeing? A systematic review and meta-analysis of mediation studies. Clin Psychol Rev..

[80] van der Velden AM, Kuyken W, Wattar U, Crane C, Pallesen KJ, Dahlgaard J (2015). A systematic review of mechanisms of change in mindfulness-based cognitive therapy in the treatment of recurrent major depressive disorder. Clin Psychol Rev..

[81] Berking M, Wirtz CM, Svaldi J, Hofmann SG (2014). Emotion regulation predicts symptoms of depression over five years. Behav Res Ther.

[82] Brockmeyer T, Kulessa D, Hautzinger M, Bents H, Backenstrass M (2015). Differentiating early-onset chronic depression from episodic depression in terms of cognitive-behavioral and emotional avoidance. J Affect Disord.

[83] Campbell-Sills L, Barlow DH, Brown TA, Hofmann SG (2006). Acceptability and suppression of negative emotion in anxiety and mood disorders. Emotion.

[84] Sharplin GR, Jones SB, Hancock B, Knott VE, Bowden JA, Whitford HS (2010). Mindfulness-based cognitive therapy: an efficacious community-based group intervention for depression and anxiety in a sample of cancer patients. Med J Aust.

[85] Michalak J, Heidenreich T, Meibert P, Schulte D (2008). Mindfulness predicts relapse/recurrence in major depressive disorder after mindfulness-based cognitive therapy. J Nerv Mental Dis..

[86] Petrocchi N, Ottaviani C (2016). Mindfulness facets distinctively predict depressive symptoms after two years: the mediating role of rumination. Personal Individ Differ.

[87] Desrosiers A, Vine V, Klemanski DH, Nolen-Hoeksema S (2013). Mindfulness and emotion regulation in depression and anxiety: common and distinct mechanisms of action. Depression and Anxiety.

[88] Nierenberg AA, Husain MM, Trivedi MH, Fava M, Warden D, Wisniewski SR (2010). Residual symptoms after remission of major depressive disorder with citalopram and risk of relapse: a STAR* D report. Psychol Med.

[89] Eisendrath SJ, Gillung E, Delucchi KL, Segal ZV, Nelson JC, McInnes LA (2016). A randomized controlled trial of mindfulness-based cognitive therapy for treatment-resistant depression. Psychother Psychosom.

[90] Kuyken W, Hayes R, Barrett B, Byng R, Dalgleish T, Kessler D, et al. Effectiveness and cost-effectiveness of mindfulness-based cognitive therapy compared with maintenance antidepressant treatment in the prevention of depressive relapse or recurrence (PREVENT): a randomised controlled trial. Lancet. 2015;386(9988):63–73. 10.1016/S0140-6736(14)62222-4. 10.1016/S0140-6736(14)62222-4 25907157

[91] Shallcross AJ, Gross JJ, Visvanathan PD, Kumar N, Palfrey A, Ford BQ, Dimidjian S, Shirk S, Holm-Denoma J, Goode KM, Cox E, Chaplin W, Mauss IB (2015). Relapse prevention in major depressive disorder: mindfulness based cognitive therapy versus an active control condition. J Consult Clin Psychol.

[92] Schwarz N, Oyserman D (2001). Asking questions about behavior: cognition, communication, and questionnaire construction. The American Journal of Evaluation.

[93] Grossman P, Van Dam NT (2011). Mindfulness, by any other name … : trials and tribulations of sati in western psychology and science. Contemporary Buddhism.

[94] Faul F, Erdfelder E, Lang AG, Buchner A (2007). G* Power 3: A flexible statistical power analysis program for the social, behavioral, and biomedical sciences. Behav Res Methods..

